# Cell-free mitochondrial DNA in human follicular fluid: a promising bio-marker of blastocyst developmental potential in women undergoing assisted reproductive technology

**DOI:** 10.1186/s12958-019-0495-6

**Published:** 2019-07-10

**Authors:** Yu Liu, Qiuzi Shen, Xue Zhao, Min Zou, Shumin Shao, Jiao Li, Xinling Ren, Ling Zhang

**Affiliations:** 1 Family Planning Research Institute and Center for Reproductive Medicine, Tongji Medical College, Huazhong University of Science and Technology, Wuhan,, 430030 People’s Republic of China; 2 Reproductive Medicine Center, Tongji Hospital, Tongji Medical College, Huazhong University of Science and Technology, Wuhan, 430030 People’s Republic of China

**Keywords:** Cell-free mitochondrial DNA, Cell-free DNA, Human follicular fluid, Oocyte quality, Age

## Abstract

**Background:**

Cell-free mitochondrial DNA (cf-mtDNA) in body fluids has attracted much attention for the purpose of monitoring disease because of the clinical advantages. This study investigated whether the cf-mtDNA content in human follicular fluid samples was associated with oocyte and embryo developmental competence.

**Methods:**

We collected 225 individual follicular fluid samples from 92 patients undergoing conventional in vitro fertilization (*n* = 53) or intracytoplasmic sperm injection (*n* = 39). cf-mtDNA and cell-free nuclear DNA (cf-nDNA) were measured using real-time quantitative PCR for the ND1 and *β*-globin genes. Multivariate logistic regression and linear regression were used to analyze data.

**Results:**

The relative cf-mtDNA content (cf-ND1/cf-*β*-globin ratio) in follicular fluid was significantly lower in the group showing blastocyst development than in the non-blastocyst group (*P* = 0.030). Additionally, the relative cf-mtDNA content was significantly and positively correlated with the age of the female patient (*P* = 0.009), while the relative cf-mtDNA content for older women (≥38 years old) with anti-Müllerian hormone (AMH) ≤1.1 ng/ml was significantly higher than in those with AMH > 1.1 ng/ml (*P* <0.05). The cf-nDNA content was significantly positively correlated with the antral follicle count (*P* = 0.012), and significantly negatively correlated with both the number of days of stimulation and the total dose of gonadotropin administration (*P* = 0.039 and *P* = 0.015, respectively). Neither cf-mtDNA nor cf-nDNA levels in follicular fluid were associated with oocyte maturation, fertilization, or Day 3 embryo morphological scoring.

**Conclusions:**

The relative cf-mtDNA content in human follicular fluid was negatively correlated with blastulation and positively correlated with the patient age, indicating that it is a promising bio-marker to evaluate oocyte developmental competence.

**Electronic supplementary material:**

The online version of this article (10.1186/s12958-019-0495-6) contains supplementary material, which is available to authorized users.

## Introduction

Oocyte quality is a crucial factor influencing embryo developmental competence and clinical pregnancy rate, but the evaluation of oocyte quality is mainly limited to an assessment of morphological criteria in most in vitro fertilization (IVF) laboratories. Several studies have identified potential bio-markers from follicular fluid and granulosa cells [1–3], but their use remains controversial and requires further validation so they cannot be used in clinical applications. Additionally, the expression levels of certain genes in cumulus cells showed promise, but these were affected by ovarian stimulation protocols and patient characteristics [4, 5].

Cell-free DNA (cf-DNA) in plasma or body fluids includes cell-free nuclear DNA (cf-nDNA) and cell-free mitochondrial DNA (cf-mtDNA). cf-nDNA is widely present in the physiological extracellular milieu, and has been found in blood, urine, saliva, spinal fluid, semen, and follicular fluid [6–12]. It is thought to have great application value in the diagnosis and prognosis of cancer and prenatal diagnosis [13–16]. Cf-mtDNA is also detected in various body fluids [10, 17, 18] and has some unique characteristics compared with nuclear DNA, including a short length, simple molecular structure, and multiple copy number.

Mitochondria are very important organelles that function as powerhouses but are also involved in numerous other cellular functions including cell proliferation, apoptosis, and intracellular calcium homeostasis [19, 20]. An aberrant amount of mtDNA can lead to mitochondrial dysfunction and the development of disease. Therefore, it is reasonable that cf-mtDNA is a promising molecular marker with high sensitivity. Indeed, accumulating evidence demonstrates that plasma or serum cf-mtDNA levels differ significantly between cancer patients and healthy individuals [21, 22] .

cf-DNA mainly derives from apoptotic cells and live cells showing active secretion [23]. Apoptotic cells release not only nuclear DNA but also mtDNA. Scalici et al studied the integrity of cf-DNA in follicular fluid and showed that around 85% of follicular fluid cf-DNA derived from cell apoptosis [8]. Another origin of cf-mtDNA is mitochondrial dysfunction. Recently, Kansaku et al reported that cultured oocyte–cumulus complexes with mitochondrial dysfunction secreted more cf-mtDNA into the medium than those with normal mitochondrial function [24].

The mitochondrial genome to nuclear genome ratio (Mt/N), assessed using real-time quantitative PCR, is often used to reflect changes in the mtDNA content, and Malik et al proposed that changes in body fluid Mt./N could be a biomarker of mitochondrial dysfunction [25]. Therefore, we inferred that cf-nDNA in follicular fluid could reflect granulosa cell apoptosis, while the relative cf-mtDNA content could reflect the change in mitochondrial function and dynamics of granulosa cells. Several studies revealed that the amount of cf-DNA in human follicular fluid was associated with the corresponding embryo quality, and could be used as a novel biomarker to predict the quality of embryos [8, 16]. However, the relationship of cf-mtDNA in human follicular fluid and oocyte and embryo developmental competence is unclear.

Therefore, in the present study, we investigated the relationship between follicular fluid cf-mtDNA levels and oocyte developmental competence and explored the effect of patient clinical characteristics on cf-mtDNA levels in follicular fluid.

## Materials and methods

### Patients’ characteristics

This research recruited 92 women enrolled in IVF (*n* = 53) or intracytoplasmic sperm injection (ICSI) (*n* = 39) cycles at the Center for Reproductive Medicine of Tongji Medical College at the Huazhong University of Science and Technology from October 2017 to July 2018. The women’s ages ranged from 21 to 45 years (mean ± SD: 32.13 ± 4.85 years) and their body mass index (BMI) ranged from 16.60 kg/m^2^ to 33.90 kg/m^2^ (mean ± SD: 22.38 ± 3.58 kg/m^2^). The duration of infertility was 4.20 ± 3.57 years, and 53% of couples had primary infertility. Female infertility had been identified in 60% couples, male factors in 23%, and mixed infertility in 17%. The number of days of stimulation ranged from 3 to 22 days (mean ± SD: 9.75 ± 2.74), and the total dose of gonadotropins received ranged from 225 to 6800 IU (mean ± SD: 2089.69 ± 835.12). Baseline hormonal levels (follicle-stimulating hormone [FSH], luteinizing hormone [LH], and 17*β*-estradiol [E_2_]) and anti-Müllerian hormone (AMH) were assessed using the Beckman DXI800 chemiluminescence analyzer (Beckman Coulter Inc., Brea, CA) on the third day of the menstrual cycle for each patient. Levels of AMH < 1.1 ng/ml are considered to reflect a reduced ovarian reserve, and levels ≥1.1 ng/ml are normal ovarian reserve [26]. Levels of FSH ≥ 9 IU/L represent a reduced ovarian reserve.

The ovarian stimulation regimens used included long agonist protocols, ultra-long agonist protocols, antagonist protocols, and progestin-primed ovarian stimulation (PPOS). Oral progestin was first given to prevent a premature LH surge in PPOS. Pituitary inhibition was performed with a gonadotropin-releasing hormone agonist or antagonist for other protocols. FSH stimulation was monitored by measuring serum E_2_ levels and follicular size. Human chorionic gonadotrophin (hCG) (Livzon, Zhuhai, China) was injected when at least three follicles reached a diameter of 18 mm or more by ultrasound inspection. Oocytes were retrieved by trans-vaginal ultrasound-guided puncture 36 h after hCG injection.

### Morphological assessment of oocytes, cleavage embryos, and blastocysts

Cumulus cells were stripped to observe the extrusion of the first polar body of the oocyte prior to ICSI (Day 0), and oocytes extruding the first polar body were called mature oocytes. For IVF oocytes, oocyte maturity was confirmed when the cumulus cells were stripped 4–6 h after IVF. Zygotes with pronuclei present 18–20 h after microinjection or insemination were considered representative of fertilization. Oocytes that underwent cleavage on Day 2 without pronuclei on Day 1 were also considered representative of fertilization for IVF. Day 3 embryos were morphologically scored in accordance with the current consensus system [27]. High-quality embryos were defined as those with at least six blastomeres of a uniform shape on Day 3, and with fragments less than 25%. One or two high-quality embryos were transferred or frozen on Day 3, and the rest were cultured to the blastocyst stage to be transferred or frozen. Blastocysts were evaluated morphologically based on the expansion of the blastocoele (3–6 stages) and the number and cohesiveness of the inner cell mass and trophectoderm (Grade A, B, and C), according to Gardner’s criteria [28].

### Follicular fluid collection and preparation

Two hundred and twenty-five follicular fluid samples without flushing media were collected from individual follicles of 92 patients, centrifuged at 3000×*g* for 15 min and 16,000×*g* for 10 min, and then immediately stored at − 80 °C until cf-nDNA and cf-mtDNA quantification. To prevent any blood pollution, only clear follicular fluid samples were involved, while bloodstained and cloudy follicular fluid samples were discarded.

### Quantification of cf-nDNA and cf-mtDNA

cf-DNA was extracted and purified from follicular fluid by the BeaverBeads™ Circulating DNA kit (BEAVER, Suzhou, China) according to the manufacturer’s instructions. cf-nDNA and cf-mtDNA in individual follicular fluid samples were estimated by amplification using *β*-globin and ND1 primers using real-time quantitative polymerase chain reaction (qPCR). Primers were designed and synthesized by Sangon Biotechnology Co., Ltd. (Shanghai, China). Primers for ND1 were 5′-CCCTAAAACCCGCCACATCT-3′ (forward) and 5′-GAGCGATGGTGAGAGCTAAGGT-3′ (reverse), which amplify a 69 bp DNA fragment. *β* -globin was detected using the following primers: 5′-AAAGGTGCCCTTGAGGTTGTC-3′ (forward) and 5′-TGAAGGCTCATGGCAAGAAA-3′ (reverse), which amplify a 77 bp DNA fragment. The amplicons were detected using primer sequences and verified in the GenBank database (Additional file 1: Figure S1). Standard curves were made using purified plasmid DNA corresponding to ND1 and *β*-globin (Additional file 1: Figure S1). The relative content of cf-mtDNA in follicular fluid was expressed by the cf-ND1/cf-*β*-globin ratio. All reactions were performed in duplicate on the StepOne™ and StepOnePlus™ Software system (ThermoFisher Scientific, Waltham, MA, USA). Reactions were performed in a total volume of 20 μl containing 2 μl of sample template (elution product of the BeaverBeads™ Circulating DNA kit), 10 μM of forward and reverse primers, and 10 μl of Hieff™ qPCR SYBR® Green Master Mix (High Rox Plus; Yeasen, Shanghai, China). Cycling conditions were as follows: 95 °C for 30 s, then 40 cycles of 95 °C for 5 s and 60 °C for 30 s.

### Statistical analysis

All measurement data were presented as means ± standard deviation (SD), or as median values and the interquartile range (IQR), if appropriate. Statistical analyses were performed using the Statistical Package for Social Sciences program, Version 12.0 (SPSS Inc., Chicago, IL, USA). Linear regression was carried out for the effect of patient clinical information on cf-nDNA and cf-mtDNA levels in follicular fluid. The Wilcoxon rank sum test was performed to compare different oocyte developmental outcomes. Multivariate logistic regression was then carried out to further characterize cf-nDNA and cf-mtDNA levels as predictors of embryo grade and blastocyst development. One way non-parametric analysis of variance (Kruskal-Wallis test) was used to analyze cf-nDNA and cf-mtDNA statistical data among four groups by age combined with AMH or FSH. Statistical significance was assumed at *P* < 0.05.

## Results

### The relationship between relative cf-mtDNA content in follicular fluid and embryo developmental competence

**Fig. 1:**
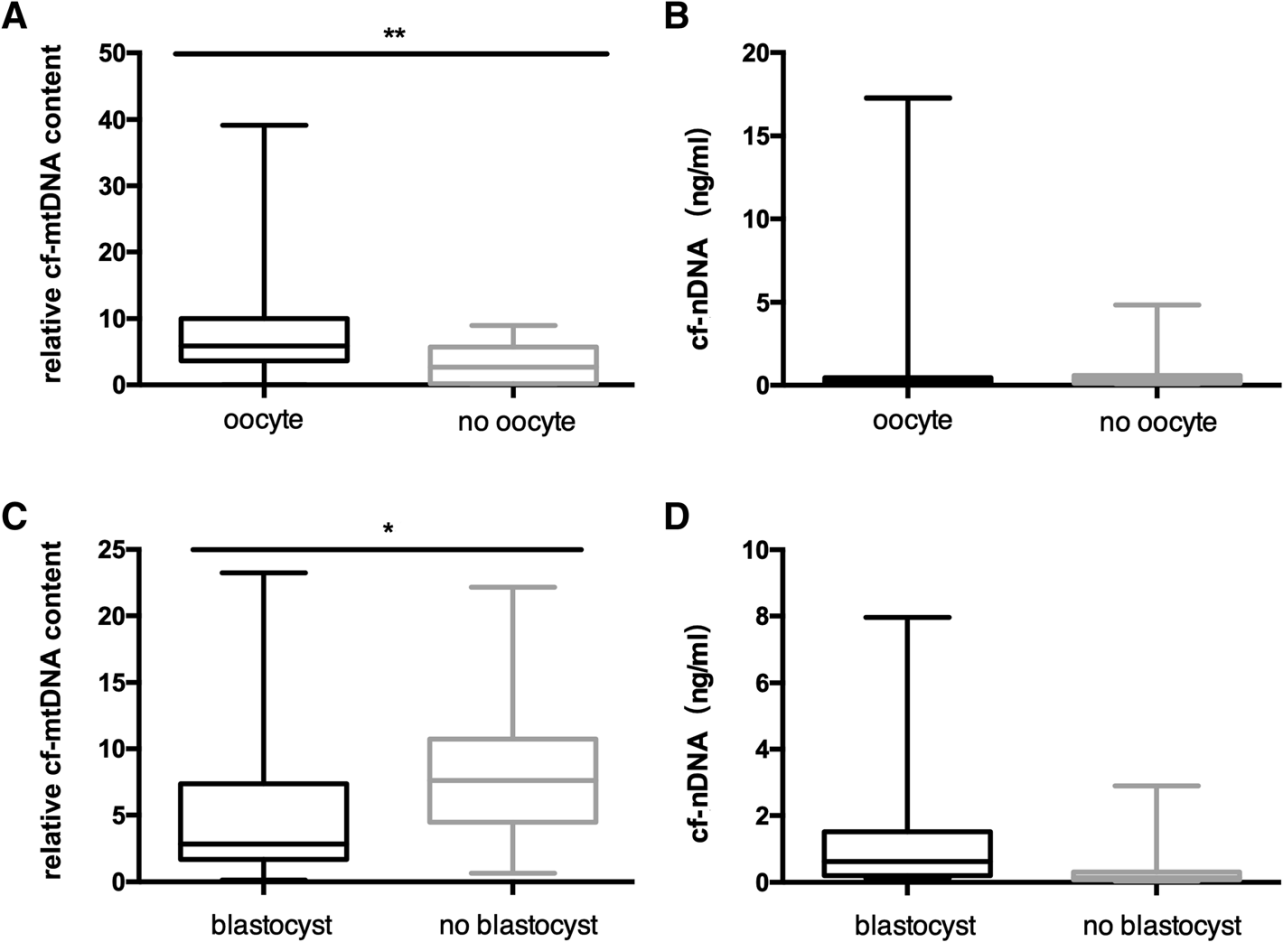
The relationship of cell-free nDNA and the relative cell-free mitochondrial DNA (cf-ND1/cf-*β*-globin ratio) levels in follicular fluids and oocyte presence and blastulation. No oocyte means that no oocyte was found in the follicular fluid. No blastocyst means that embryos did not develop into the blastocyst stage. *P < 0.05, **P < 0.01

Of 225 individual follicular fluids, a total of 190 had mature oocytes (84%), 17 had immature oocytes (8%), 16 had no oocytes (7%), one had a degenerated oocyte, and one was naked without a zona pellucida. The relative cf-mtDNA content (cf-ND1/cf-*β*-globin ratio) was significantly higher in follicular fluids with than without oocytes (*P* < 0.01; Fig. 1a). Because the criteria for oocyte maturation and fertilization differ between IVF and ICSI, we carried out statistical analysis for IVF and ICSI oocytes separately (Table 1). The relative cf-mtDNA content did not differ between mature and immature oocytes, or between fertilized and non-fertilized oocytes for IVF or ICSI (*P* > 0.05).

**Table 1:** the cf-nDNA and the relative cf-mtDNA (cf-ND1/cf-*β*-globin ratio) levels in human follicular fluids for oocytes performing IVF or ICSI

	Total			IVF			ICSI		
FF associated with	n of FF(%)	cf-nDNA MD (IQR)	relative cf-mtDNA MD (IQR)	n of FF(%)	cf-nDNA MD (IQR)	relative cf-mtDNA MD (IQR)	n of FF(%)	cf-nDNA MD (IQR)	relative cf-mtDNA MD (IQR)
Mature oocytes (MII)	190 (91.79)	0.24 (0.13–0.48)	5.70 (3.66–9.97)	118 (91.47)	0.21 (0.11–0.53)	5.80 (3.90–9.40)	72 (92.31)	0.25 (0.14–0.36)	5.56 (3.23–10.05)
Immature oocytes (MI or GV)	17 (8.21)	0.13 (0.07–0.30)	6.50 (4.82–11.18)	11 (8.53)	0.19 (0.06–0.29)	6.51 (3.88–14.86)	6 (7.69)	0.12 (0.09–0.13)	6.63 (5.03–7.83)
Fertilized oocytes	170 (81.34)	0.23 (0.13–0.46)	6.06 (3.90–10.01)	106 (80.92)	0.21 (0.11–0.50)	6.51 (4.02–10.01)	64 (82.05)	0.25 (0.14–0.38)	5.46 (2.99–10.01)
Unfertilized oocytes	39 (18.66)	0.18 (0.08–0.37)	5.57 (3.72–10.16)	25 (19.08)	0.20 (0.07–0.50)	5.11 (2.88–10.23)	14 (17.95)	0.13 (0.09–0.26)	7.78 (4.94–9.97)

Data are presented as MD (IQR). Percentages are calculated within groups. *FF* follicular fluid. *MI* metaphase I, *MII* metaphase II, *GV* germinal vesicle, *MD* Median, *IQR* interquartile range

According to cleavage embryo evaluation criteria, 89 (89/142, 63%) embryos were of high quality and 53 (53/142, 37%) were poor quality. There was no significant difference in the relative cf-mtDNA content between high-quality and poor-quality embryos (*P* > 0.05). In our samples, 43 embryos were cultured until Day 6. Twenty-two (51%) embryos developed into blastocysts, and 21 (49%) embryos arrested or failed to develop on Day 5 or Day 6. The relative cf-mtDNA content in the blastocystgroup was significantly lower than in the non-blastocyst group (2.84 versus 7.80; *P* = 0.030; Fig. 1c and Table 2).

**Table 2:** Relationship of the cf-nDNA and the relative cf-mtDNA (cf-ND1/cf-*β*-globin ratio) levels in human follicular fluid with embryo development outcomes

FF associated with	n of FF(%)	cf-nDNA (ng/ml)			relative cf-mtDNA		
		MD (IQR)	OR (95% CI)	*p*	MD (IQR)	OR (95% CI)	*p*
High-quality embryos	89 (62.68)	0.23 (0.13–0.50)	0.94 (0.78, 1.12)	0.46	5.27 (2.99–9.67)	1.00 (0.95, 1.05)	0.94
poor-quality embryos	53 (37.32)	0.24 (0.13–0.32)			7.39 (4.14–10.07)		
blastocysts	22 (51.16)	0.63 (0.21–1.81)	26.43 (0.75, 929.72)	0.071	2.84 (1.53–6.99)	0.87 (0.76, 0.99)	0.030
No blastocysts	21 (48.84)	0.14 (0.08–0.25)			7.80 (4.66–10.34)		

Percentages are calculated within groups. Data are presented as MD (IQR) and are analyzed using multivariate logistic regression. Statistical significance was assumed at *P* < 0.05. *FF* follicular fluid, *MI* metaphase I, *MII* metaphase II, *GV* germinal vesicle, *OR* odds ratio, *CI* confidence interval, *MD* Median, *IQR* interquartile range

### Effect of patient characteristics on the relative cf-mtDNA content in follicular fluid

**Fig. 2:**
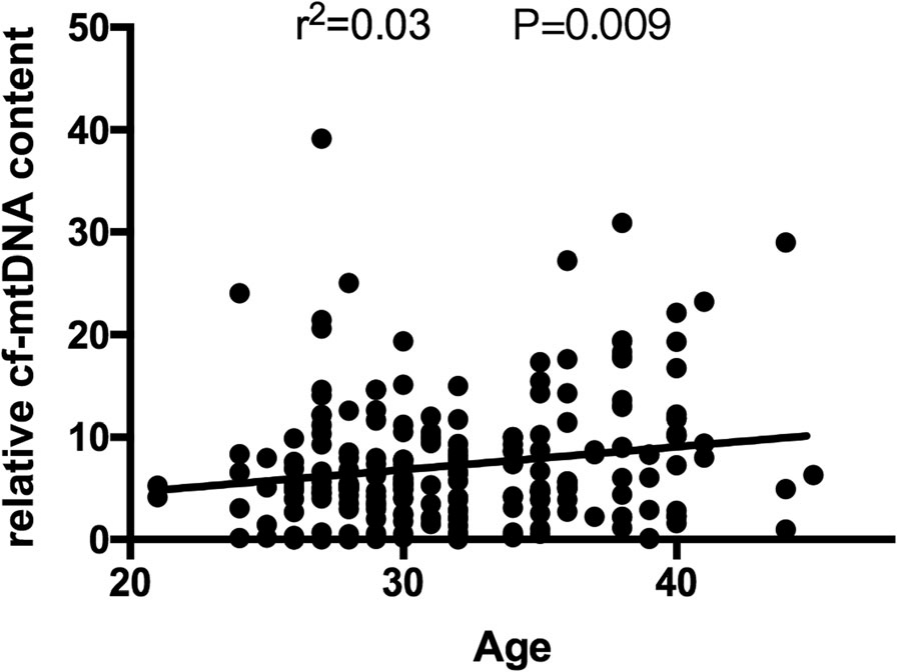
Correlations between the relative cf-mtDNA content (cf-ND1/cf-*β*-globin ratio) in follicular fluid and patient age

The relative cf-mtDNA content in follicular fluid correlated significantly positively with age (*β* ± SE: 0.22 ± 0.085, *P* = 0.009) (Table 3; Fig. 2) but not with AMH, basal FSH level, or antral follicle count (AFC).

**Table 3:** Patients’ characteristics association with the cf-nDNA and the relative cf-mtDNA (cf-ND1/cf-*β*-globin ratio) levels in individual human follicular fluid

Variable	Mean	n (%)	Min-Max	SD	cf-nDNA		relative cf-mtDNA	
					***β*** ± SE	***p***	***β*** ± SE	***p***
Age (years)	32.13	225 (100)	21–45	4.85	−0.034 ± 0.03	0.25	0.22 ± 0.085	0.009
BMI (kg/m2)	22.38	225 (100)	16.6–33.9	3.58	0.069 ± 0.044	0.12	−0.15 ± 0.13	0.25
AMH (ng/ml)	3.37	219 (97.3)	0.33–8.67	1.99	0.0007 ± 0.075	0.70	0 ± 0.20	0.89
Basal FSH (IU/L)	7.49	225 (100)	1.79–17.52	2.60	0.025 ± 0.057	0.66	−0.27 ± 0.16	0.09
Basal LH (IU/L)	4.45	225 (100)	0.51–21.41	2.49	0.001 ± 0.059	0.25	0.016 ± 0.17	0.61
Basal E_2_ (pg/ml)	43.56	225 (100)	6–299.8	39.24	0 ± 0.004	0.88	0.006 ± 0.010	0.26
Antral follicle count	17.48	225 (100)	3–40	7.84	0.026 ± 0.017	0.012	−0.050 ± 0.053	0.34
Days of stimulation	9.75	225 (100)	3–22	2.74	−0.019 ± 0.05	0.039	0.0023 ± 0.15	0.48
Total dose of gonadotropins (IU)	2089.69	225 (100)	225–6800	835.12	−0.027 ± 0.0002	0.015	0.0014 ± 0.0005	0.58
Ultra-long protocol	–	97 (43.11)	–	–				ref
Long protocol	–	17 (7.56)	–	–	0.054 ± 0.57	0.96	−1.56 ± 1.64	0.34
Antagonist protocol	–	71 (31.56)	–	–	0.17 ± 0.34	0.63	−0.18 ± 0.98	0.86
PPOS	–	40 (17.78)	–	–	−0.37 ± 0.41	0.36	1.33 ± 1.17	0.26

Data are presented as means ±standard deviation (SD). *β* ± SE, regression coefficient ± standard error. *P*-values is the result of linear mixed models. Statistical significance was assumed at *P* < 0.05.BMI, body mass index. *FSH* follicle-stimulating hormone, *LH* luteinizing hormone, *E*_*2*_ 17*β*-estradiol, *AMH* anti-Müllerian hormone, *PPOS* progestin-primed ovarian stimulation

**Fig. 3:**
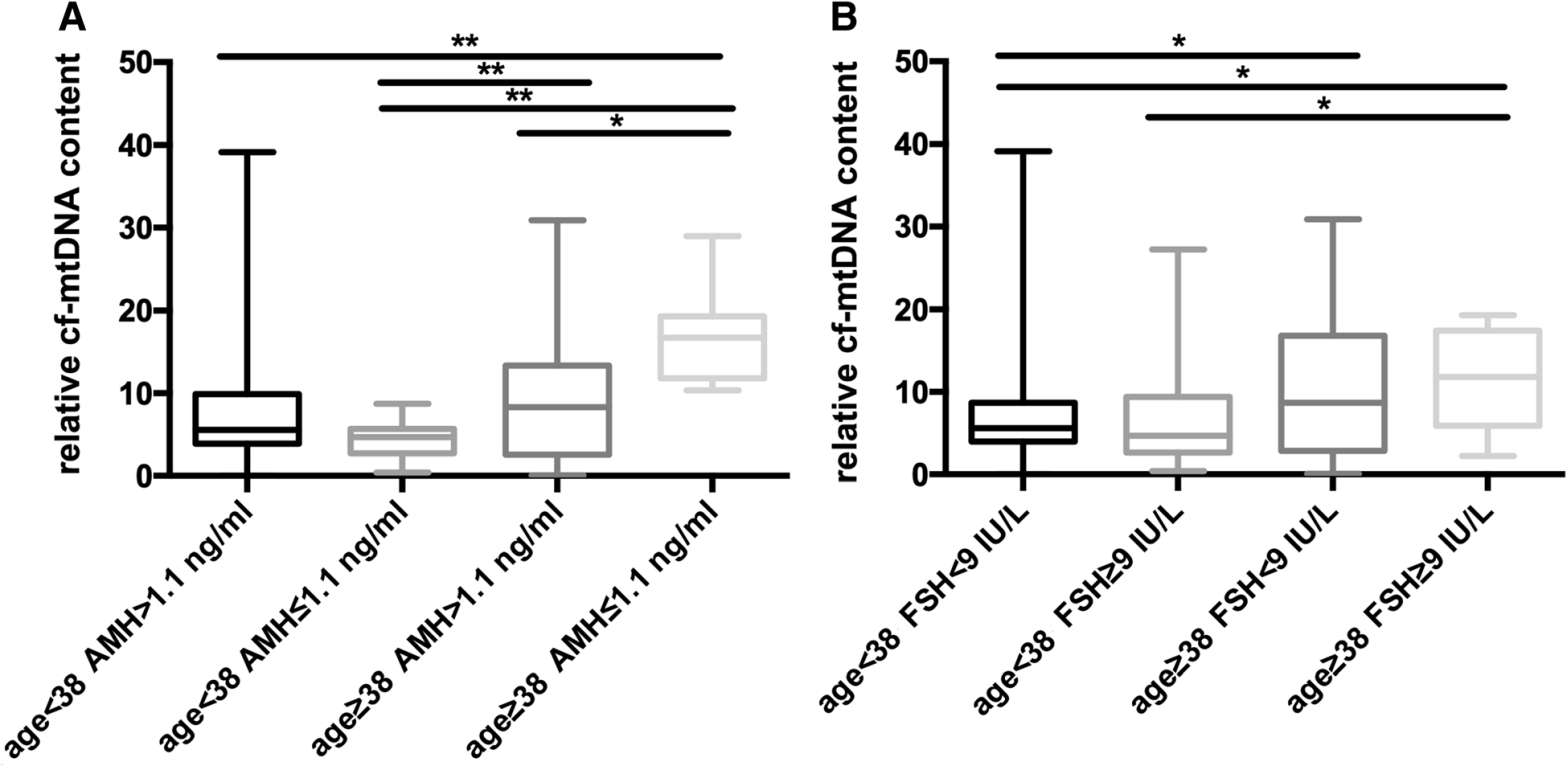
Effect of patient age combined with AMH or FSH on the relative cf-mtDNA content (cf-ND1/cf-*β*-globin ratio) in human follicular fluid. *P < 0.05, **P < 0.01

Patients were divided into four groups according to age and AMH level: age ≥ 38 years old and AMH > 1.1 ng/ml, age ≥ 38 years old and AMH ≤1.1 ng/ml, age < 38 years old and AMH > 1.1 ng/ml, and age < 38 years old and AMH ≤1.1 ng/ml. The relative cf-mtDNA content in the two groups of age ≥ 38 years old was significantly higher than that in both age < 38 years old groups (*P* < 0.05; Fig. 3a). Moreover, for women aged ≥38 years old, the relative cf-mtDNA content when AMH ≤1.1 ng/ml was significantly higher than when AMH > 1.1 ng/ml (*P* < 0.05; Fig. 3a). Similar results were observed for patient age and FSH levels (Fig. 3b).

The relative cf-mtDNA content in follicular fluid was not associated with BMI, the ovarian stimulation regimen, number of days of stimulation, or the total dose of gonadotropins (Table 3).

### Relationship of the cf-nDNA level in follicular fluid and embryo developmental competence

The median quantity and the IQR of cf-nDNA levels measured by *β* -globin qPCR in 225 human follicular fluid samples from individual follicles was 0.23 ng/ml (IQR: 0.12–0.46 ng/ml). cf-nDNA levels did not differ in follicular fluids with and without oocytes (*P* > 0.05; Fig. 1b). cf-nDNA levels in follicular fluids were not associated with oocyte maturation, fertilization, or Day 3 embryo morphological scoring. cf-nDNA levels were higher in follicular fluids where the oocytes were fertilized and reached the blastocyst stage than in those with no blastocyst development (0.63 ng/ml versus 0.14 ng/ml), but the difference was not significant after multivariate logistic regression (*P* = 0.071; Fig. 1d; Table 2).

### Effect of patients’ clinical characteristics on cf-nDNA levels in follicular fluids

There was no significant correlation between cf-nDNA levels in individual follicular fluid samples and patient age, BMI, AMH, basal hormone (basal FSH, LH, and E_2_), or the ovarian stimulation regimen (*P* > 0.05). The AFC was significantly positively correlated with the amount of cf-nDNA in follicular fluid (*β* ± SE: 0.026 ± 0.017; *P* = 0.012; Table 3). Moreover, both the number of days and total dose of gonadotropin administration significantly and negatively affected cf-nDNA levels in follicular fluid (*β* ± SE: − 0.019 ± 0.05 and − 0.027 ± 0.0002, respectively; *P* = 0.039 and *P* = 0.015, respectively; Table 3).

## Discussion

In the present study, we showed for the first time that the relative cf-mtDNA content (cf-ND1/cf-*β*-globin ratio) in human individual follicular fluid was associated with the corresponding potential of oocytes that had developed to the blastocyst stage. Moreover, it was also found to be positively correlated with patient age.

A key factor of poor oocyte quality in older women is mitochondrial dysfunction [19]. Several studies revealed a significant decrease in the mtDNA of oocytes and cumulus cells in older women compared with young women [19, 29]. Our work indicated that the relative cf-mtDNA content in follicular fluid of older women was much higher than in younger women. Moreover, in older women, the relative cf-mtDNA content in follicular fluid when AMH was > 1.1 ng/ml was significantly lower than in those with AMH < 1.1 ng/ml. We inferred from this that mitochondrial dysfunction in older women led to an increase of cf-mtDNA in follicular fluid and a decrease of mtDNA in oocytes and cumulus cells. Mitochondria are major determinants of oocyte developmental competence. Thus the relative cf-mtDNA level in follicular fluid could reflect oocyte quality. Our result demonstrated that the relative cf-mtDNA content in the follicular fluid of the group showing blastocyst development was significantly lower than in the non-blastocyst group (*P* = 0.030) using multivariate logistic regression. Intriguingly, the relative cf-mtDNA content was not affected by BMI, the ovarian stimulation regimen, or the days and doses of ovarian stimulation. Therefore, the relative cf-mtDNA content of follicular fluid is a more promising bio-marker than the expression of certain genes in cumulus cells in assessing oocyte developmental competence.

The relative cf-mtDNA content was not associated with oocyte fertilization or the cleavage embryo score. Sirard et al proposed that oocyte developmental competence included five separate events: the ability to resume meiosis, cleavage upon fertilization, development into a blastocyst, the induction of pregnancy, and the generation of healthy offspring [30]. The ability to develop into a blastocyst is the most crucial marker of oocyte competence; it is readily determined so is widely used by most laboratories, and is of use because a blastocyst has more chance of inducing pregnancy than a cleaved embryo [31]. By contrast, embryo morphological scoring based on static observations only is considered a limited method of evaluating embryo quality [32]. In the present study, we observed a low number of embryos undergoing blastocyst culture, so additional studies of larger sample sizes are needed to further confirm the relationship between the cf-mtDNA content in follicular fluid and blastulation and embryo implantation.

The cf-nDNA level measured by *β*-globin was not associated with the cleavage embryo grade. Conversely, Scalici et al and Traver et al indicated that cf-DNA in human follicular fluid was associated with embryo score and the extent of fragmentation [8, 33]. This discrepancy may arise from differences in cf-DNA extraction and quantitative methods. For example, the earlier studies did not extract cf-DNA from follicular fluid, while we used a technique based on magnetic beads. Although DNA extraction and purification steps may result in a loss of DNA, the elimination of these steps may cause components in serum to inhibit the PCR efficiency. Additionally, we used *β*-globin primers to quantify cf-nDNA, while the earlier studies used ALU115 primers. ALU is the most abundant interspersed repeated sequence in the human genome and is found at a copy number of ∼1.4 × 10^6^ per genome [34]. By contrast, the *β*-globin gene is a single copy sequence of the human genome that is commonly used in the quantitative analysis of cf-nDNA [35, 36]. We chose to amplify it in our study because we wished to determine the Mt/N ratio so the amplification of a single copy sequence was more suitable.

It is also notable that we detected higher cf-nDNA levels in follicular fluid corresponding to blastocyst development than in that without blastocysts though the difference was not significant (*P* = 0.071). Cf-DNA in follicular fluid mainly derives from apoptotic granulosa cells [8] . Our results appear to suggest that the high cf-nDNA level, or high apoptotic level of granulosa cells was a reflection of good quality oocyte, which is not in agreement with previous work that revealed an increased level of granulosa cell apoptosis in older women that was associated with a decline in oocyte quality [37]. However, previous results were affected by many confounding factors and methods of apoptotic evaluation. Recently, Regan et al proposed that granulosa cell apoptosis was an integral part of normal follicle development that varied in a stage-dependent manner [38]. Two critical stages of granulosa cell apoptosis are dominant follicle selection and preovulatory maturation. Both stages are observed at higher levels in younger compared with older women [38, 39]. Because follicular fluid samples collected during oocyte retrieval in the present study were only obtained from the preovulatory stage, it is conceivable that the low cf-nDNA level detected was a reflection of poor quality oocyte in patients.

We also found that AFC was positively correlated with cf-nDNA levels in follicular fluid, and both the number of days and total gonadotropin dose were negatively linked with cf-nDNA levels in follicular fluid, which are in agreement with the above result. AFC is a known indicator of ovarian reserve. High FSH doses were previously associated with a decrease in the number of transferable embryos and live births [40, 41]. We therefore inferred that higher cf-nDNA levels in the follicular fluid of preovulatory follicles represented normal follicular development and oocyte quality. Further study is needed to clarify this relationship.

## Conclusion

The current study showed that changes in the relative cf-mtDNA content of human follicular fluid correlate with blastocyst developmental potential and patient age, suggesting that the relative cf-mtDNA content has potential use in evaluating oocyte and embryo developmental competence. The cf-mtDNA and cf-nDNA cannot be separated and detected accurately on the condition of current technologies. A deeper understanding of the mechanism underlying cf-mtDNA origin and existence forms in human follicular fluid helps to find a more accurate method to detect cf-mtDNA amount, and promote the clinic application of cf-mtDNA in the future.

## Additional file

Additional file 1: ND1 (A, B) and β-globin (C, D) recombinant plasmid sequencing and BLAST analysis and standard curves. (TIF 3071 kb)

## Data Availability

The main data of this study can be directly requested from the corresponding author.

## Abbreviations

AFC: Antral follicle count
AMH: Anti-Müllerian hormone
BMI: Body Mass Index
Cf-mtDNA: Cell-free mitochondrial DNA
Cf-nDNA: Cell-free nuclear DNA
E_2_: 17*β*-estradiol
FF: Follicular fluid
FSH: Follicle stimulating hormone
GnRH: Gonadotropin releasing hormone
HCG: Human chorionic gonadotrophin
PPOS: Progestin-primed ovarian stimulation
