# Supplementary figures and images for: Cell-free mitochondrial DNA in human follicular fluid: a promising bio-marker of blastocyst developmental potential in women undergoing assisted reproductive technology
Source: Reprod Biol Endocrinol. 2019 Jul 10;17:54. doi: 10.1186/s12958-019-0495-6 (PMC6621940; doi:10.1186/s12958-019-0495-6)

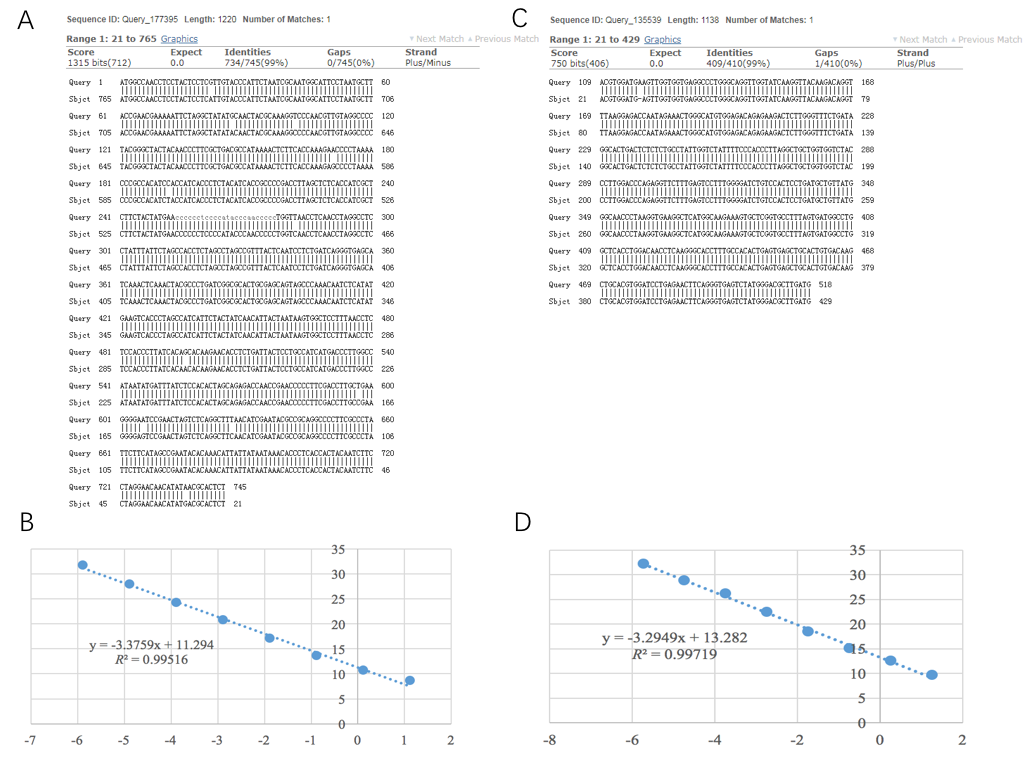

Supplement: Supplementary file 1 — ND1 (A, B) and β-globin (C, D) recombinant plasmid sequencing and BLAST analysis and standard curves. (TIF 3071 kb) [file 12958_2019_495_MOESM1_ESM.tif]
